# Evidence for Citation Networks in Studies of Free-Roaming Cats: A Case Study Using Literature on Trap–Neuter–Return (TNR)

**DOI:** 10.3390/ani10060993

**Published:** 2020-06-06

**Authors:** Michael C. Calver, Patricia A. Fleming

**Affiliations:** Environmental and Conservation Sciences, Murdoch University, Murdoch WA 6150, Australia; T.Fleming@murdoch.edu.au

**Keywords:** TNR, citation bias, citation network

## Abstract

**Simple Summary:**

All aspects of an argument need to be evaluated in evidence-based scientific pursuit to prevent the development of self-reinforcing cohorts of studies through the cross-referencing of selected subsets of studies (citation bias). Controling populations of unowned cats using Trap–Neuter–Return and its variants (hereafter TNR) is an example of how a scientific topic develops and is communicated academically and publicly. We found 145 TNR publications between 2002–2019, with the last two years seeing a rapid increase in studies. Publication clusters occur, focusing on: population control, interactions with wildlife, disease transmission (including implications for pet cats, wildlife and humans), free-roaming cats, and feral and domestic cat management. A quarter of all studies were published in Open Access formats, which have the highest potential social impact because they are free to download, share and read. While it is encouraging that diverse views are expressed, the development of clusters of introspective topics risks reduced dialogue between groups of authors with differing views. Journal editors could encourage exchanges between groups by choosing reviewers from different camps to assess manuscripts and by asking authors to acknowledge alternative views.

**Abstract:**

Trap–Neuter–Return and its variants (hereafter TNR) aims to control unowned cat populations. Papers on this topic form a useful case study of how how an area of literature grows, papers become influential, and citation networks form, influencing future study as well as public perceptions of the science. We analysed 145 TNR studies published 2002–2019. Common topics, identified by frequently used language, were population control, interactions with wildlife, disease transmission (including implications for pets, wildlife and humans), free-roaming cats, and feral and domestic cat management. One or more papers on each of these topics was judged influential because of high citations overall, high average citations/year, or frequent mentions in social media. Open Access papers were more influential in social media, raising greater public awareness than studies published in journals that were less accessible. While divergent views exist on a range of topics, the network analysis of the TNR literature indicated potential for forming self-reinforcing groups of authors. While it is encouraging that diverse views are expressed, there is a risk of reduced dialogue interactions between groups, potentially constraining dialogue to refine arguments, share information, or plan research. Journal editors could encourage communication by choosing reviewers from different camps to assess manuscripts and by asking authors to acknowledge alternative views.

## 1. Introduction

Scientific pursuit should aim to be evidence-based (e.g., [1,2,3]). Such an approach would increase assurance that scientific outcomes are reliable and rigorously tested, with scientists willing to change their views in the light of evidence. This approach was well expressed by Darwin: *‘I have steadily endeavoured to keep my mind free so as to give up any hypothesis, however much beloved (and I cannot resist forming one on every subject), as soon as facts are shown to be opposed to it.’* [4]. However, even scientists are ‘human’ and, like others, may not be prepared to shift their position regardless of evidence: *‘Scientists, like everyone else, find it difficult to accept that which does not fit their beliefs, irrespective of the evidence’* ([5] p.480). Consequently, across diverse fields of science, the published literature contains biases against citation of negative studies that do not support an established hypothesis (e.g., [6,7,8]), with selective interpretation of literature to support a particular view [9]. There is also spin in scientific communication to the public. Social media permit scientists to communicate directly to non-specialists, publicising new findings without going through the peer-review process (e.g., Chapter 9 of [10]); both information and misinformation can spread rapidly via social media, unimpeded by editorial gatekeeping. Social media are also susceptible to activism that may distort public understanding of a sound scientific message or disrupt scientifically based policy [11,12]. For example, Groshek, Basil, Guo, Ward, Francis and Jason [13] noted in their study of public understanding of inflammatory bowel disease in the United States that people posting online frequently regarding the condition were less knowledgeable about it than those who posted less often, which is a specific case of the more general principle that less competent people may have inflated perceptions of their own skill [14]. Such biases therefore mean that both scientific and public communications can sometimes ignore or distort the interpretation of facts. 

The management of unowned domestic cats by Trap–Neuter–Return and its variants (hereafter collectively together referred to as TNR) provides a case study of how knowledge, understanding and authority develop in a scientific field and are transferred to wider public understanding via social media. In TNR, unowned, free-roaming cats are trapped, desexed, sometimes vaccinated or provided with other health care, and (health permitting) preferentially returned to colonies (that may or may not include other cats) with supplemental feeding sometimes provided. The ultimate aim of such programs is to reduce or eliminate populations of unowned cats with minimal use of euthanasia, thereby mitigating the problems they cause. The topic is important because free-roaming cats, owned or otherwise, may hunt or otherwise disturb wildlife [15,16], spread disease to wildlife, pet cats or humans [17,18], hybridise with native felids in some locations [19], cause general nuisance by fighting, spraying and fouling gardens [20], and suffer poor welfare outcomes themselves through untreated trauma or ingestion of hazardous substances [21]. Thus measures to reduce or eliminate unowned cat populations are undertaken globally, with due attention to the specific needs and wishes of individual communities (e.g., [22]). 

Since 2002, there has been a growing scientific literature on TNR, covering population turnover and size (e.g., [23,24,25]), animal health and welfare (e.g., [21,25]), as well as human involvement with TNR colonies (e.g., [26,27]). The literature has grown into one of contrasting viewpoints (e.g., [28,29,30,31] cf. [32,33]. Over the past decade, there have been several reviews of the TNR literature with a focus on the effectiveness, practicability and humaneness of TNR (e.g., [28,30,33,34,35,36]); however, to our knowledge, there has been no bibliometric study of the TNR literature. Bibliometric study can indicate the extent of information exchange between authors with a strong focus on the environmental impacts of free-roaming cats (i.e., interactions with wildlife) and authors addressing cat welfare. Bibliometric study also has the potential to illuminate the social and policy significance of research and commentary on TNR and can inform how research findings should be synthesised into management of unowned cats [37,38]. We therefore aimed to:

1. Describe citation networks in the TNR literature based on text data from titles and abstracts, shared references between TNR papers in different journals, and the bibliographic coupling of authors (i.e., shared by mutual citation).
2. Describe the TNR literature in terms of the increasing number of studies over time, the journals in which the studies were published, the countries where the authors were based, the institutional affiliations of the authors (academic, government, non-government organisation, private researcher) and identify the most influential papers based on citations and on non-citation altmetrics.
3. Document the topics of major concern in the TNR literature, and test whether the relative proportions of positive, neutral or negative assessments of TNR vary depending on the topic under discussion.
4. Document the role of Open Access publication (in which papers, once published, are free to read and disseminate) in distributing TNR findings.

We specifically did not aim to evaluate papers critically nor to pass judgement on the applicability or otherwise of TNR in any situation. 

## 2. Materials and Methods 

### 2.1. Literature Search

We searched the Web of Science Core Collection (WoSCC) (coverage since 1900) on 13 January 2020 using the search terms ‘trap neuter release’, OR ‘trap neuter return’, OR ‘trap neuter vaccinate’, to locate relevant studies. While WoSCC is a highly selective database [39] requiring journals to meet 24 quality criteria and four impact criteria for inclusion [40], we reasoned that this selectivity would ensure that we were considering peer-reviewed contributions from respected journals. 

### 2.2. Identification and Description of Citation Networks

We used VOSviewer version 1.6.8 (https://www.vosviewer.com/) to visualise connections between TNR publications [41,42]. In the visualisations, items such as authors, keywords or journals are represented by a label and a circle; the greater the weight of an item (e.g., the greater number of citations to a reference/author), the larger the circle representing the item (e.g., the reference/author). Items are connected by links, which can be indicated by factors such as common publications in reference lists of papers, co-authorship links between researchers, and common use of keywords. Links have a strength of 1 or greater depending on the factor in question (e.g., a link may indicate the number of co-authored papers between two authors) with higher values indicating greater strength. A network comprises all items and the links between those items. Clusters of items appear as different colours, with connecting lines representing linkages through common usage; the thickness of connecting lines indicates the strength of the link. 

We focused on: common text data in the title and abstract fields of papers (an indication of important research questions/topics), co-citations for journals (indicating which journals are commonly co-cited together), co-authorship for all authors, and co-authorship for authors with at least two publications in the network (indicating which authors tend to be cited together).

### 2.3. Journal and Authorship Analysis

We listed the journals in which relevant papers were published, noting whether or not the journal was Open Access (OA) or, if not, whether individual papers were published OA in subscription journals. We also tallied the number of papers published in each journal. 

All authors on each paper, not just the first author, were grouped into one of three broad affiliation categories by examining the address lines in the papers: non-government organisation or private researcher (hereafter NGO), government (address indicated a government agency or department) and academia (a university or college address). If an author had multiple affiliations, only the first mentioned affiliation was entered. To assess possible changes in the distribution of authors in different affiliation categories over time, we grouped papers into four time periods- (2002–2006, 2007–2011, 2012–2016, and 2017–2019) and tested for any association between author affiliations and time period using a chi-square contingency table with expected values calculated assuming an equal proportion of the overall distribution of the categories represented across each time period. 

We also noted the country named in the address lines of all authors. If an author indicated two countries, we included only the first-named. To assess changes in the international profile of the TNR literature over time, we grouped papers into the same time periods as used for assessing author affiliations. Within those periods, we noted the distribution of countries of authors, as well as the number of ‘international’ papers, defined as those having authors from more than one country. We tested for any association between author countries and time period using a chi-square contingency table with expected values calculated assuming an equal proportion of the overall distribution of authors publishing from each country were represented across each time period.

### 2.4. Influential Publications

Influential publications were first assessed using conventional citation statistics taken directly from the WoSCC and included the proportions of papers cited over time, the 10 most highly cited papers overall, and the 10 most highly ranked papers based on citations/year since the paper was published (e.g., [43,44,45,46]). 

We also recorded alternative metrics for each study using PlumX Metrics [47], available within the Scopus database. PlumX Metrics record data on usage (clicks, downloads, etc.), captures (bookmarks, favourites, etc.), mentions (news media, blogs, reviews, etc.) and social media (shares, likes, comments, tweets, etc.). While these are not reduced to a single score as is the case with the alternative Altmetrics [48,49], PlumX Metrics are available over the year range of publication for the TNR literature. For each paper identified in our original WoS search, we summed the scores in each of the two categories in Scopus–mentions and social media–as the best indication of broad community interest. These data were collected in January 2020 for all papers detected in the literature search in WoSCC. 

### 2.5. Specific Topics of TNR Studies and Assessments of TNR Utility

Based on our own readings of the papers identified in the literature search, we grouped the papers into nine broad topic areas. We also scored all papers according to whether they took a positive, neutral or negative attitude to the use of TNR (‘stance taken’). Examples of key phrases paraphrased from papers that characterised these stances included ‘data support TNR’ (positive), ‘the evidence contradicts TNR claims’ (negative) and ‘studies provide at best only qualified support’ (neutral). We tested for associations between the topic and stance using a chi square test with expected values calculated assuming an equal proportion of the overall distribution of stances recorded were represented across each topic (negative and neutral studies combined to reduce the number of cells with small expected values and meet the criterion of a mean expected frequency ≥ 6 across all cells for a valid contingency table analysis at 0.05; [50], p. 503). Combining all studies, we also tested for an association between stance taken and whether or not papers were published OA using a two-tailed Fisher exact test. 

## 3. Results

### 3.1. Literature Search

**Figure 1:**
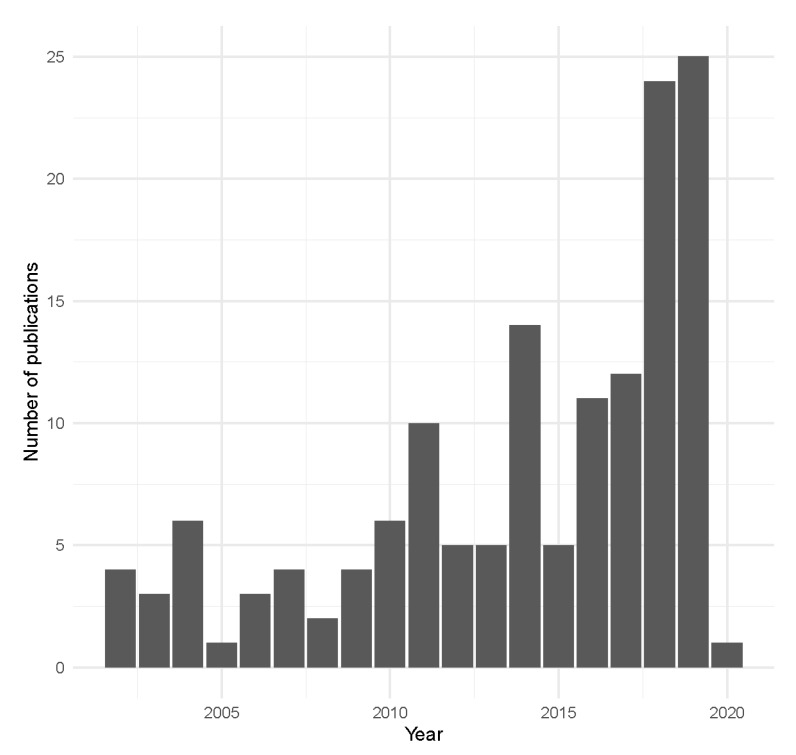
Number of publications contributing to the literature for Trap-Neuter-Release (TNR) management of cats (*Felis catus*) published each year, 2002–2019. Note that the 2020 record only includes a single publication entered in the Web of Science Core Collection at the search date of 13 January 2020.

The search in WoSCC retrieved 162 papers published between 2002 and January 2020. We excluded 17 documents that were short letters, corrections or editorial material, as well as two that pertained solely to dog management. For the 145 papers remaining, the distribution of papers across years showed a rapid increase from four in 2002 to 10 in 2011, 24 in 2018 and 25 in 2019 (Figure 1). The rate of accumulation of papers shows no sign of slackening, with further papers already published in 2020. Two journals had special editions on unowned cat management for publication in 2019, which doubled the numbers of TNR publication over the last two years.

### 3.2. Identification and Description of Citation Networks

**Figure 2:**
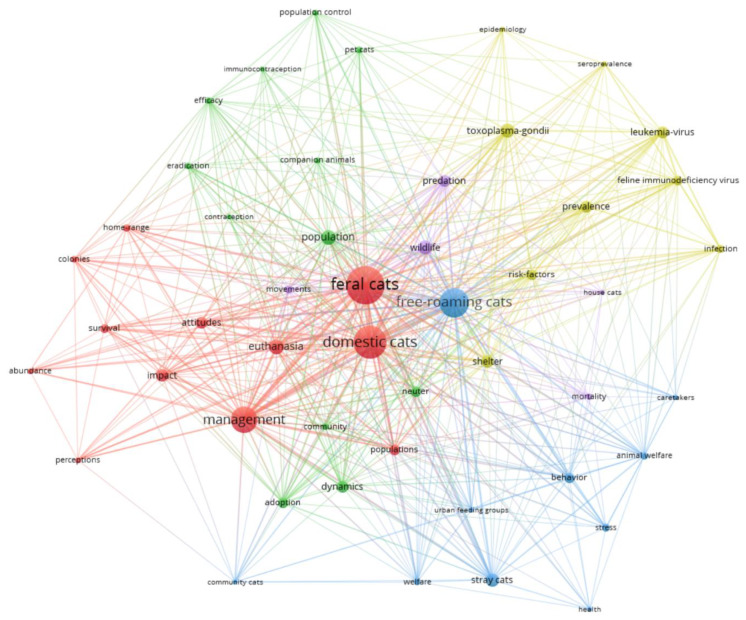
Networks of co-occurrence of 51 key terms across 145 papers published on TNR cat management. Here, each node (circle) represents one of 51 key terms that appear four or more times in the title, abstract or keyword fields for these publications. The size of the nodes represents the frequency of occurrence for each term, and relative thickness of the lines represent the incidence co-occurrence between terms.

Of the 684 terms identified in the titles, abstracts or keyword lists in the 145 papers, 51 terms appeared at least four times and were deemed relevant (i.e., were not generic or synonyms of another term) (Figure 2). They were grouped into five clusters based on the co-occurrence of words (clockwise from the top): 1. disease and health (olive), 2. stray and free-roaming cats (blue), 3. feral and domestic cat management (red), 4. population control (green), and 5. wildlife and predation (purple). The largest nodes occurred mainly in the red cluster, where ‘feral cats,’ ‘domestic cats’ and ‘management’ were prominent terms. ‘Free-roaming cats’ was the largest node in the blue cluster.

**Figure 3:**
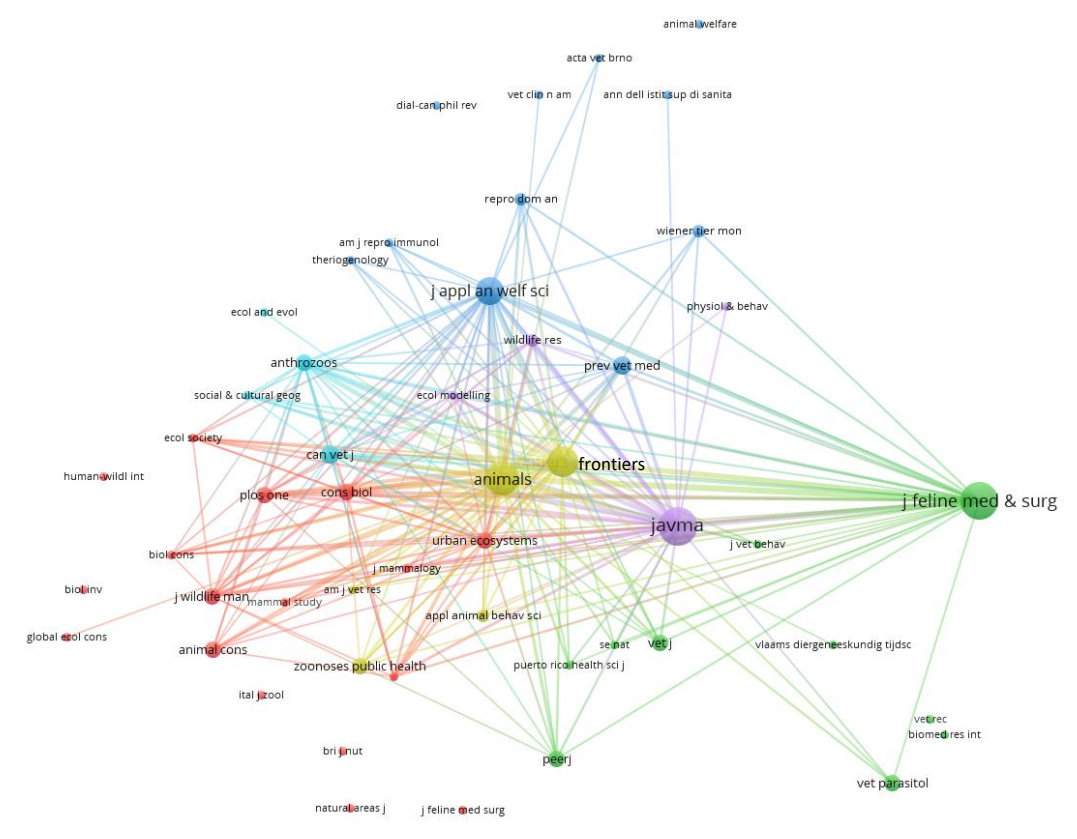
Networks for 145 papers published on TNR cat management showing bibliographic coupling links between publications in 55 journals. Here, each node (circle) represents one of 55 journals, with the Canadian Veterinary Journal and the Canadian Journal of Veterinary Research appearing as a single node as classified by the WoSCC journal abbreviation. Three papers published in *Veterinary Dermatology, Revista Medica de Chile*, and *Journal of Veterinary Internal Medicine* are not shown as they were off-scale of this image (and showed minimal connections). The size of the nodes represents the numbers of papers published in each journal, and the thickness of the lines represents the strength of bibliographic coupling, where the strength is determined by the number of shared references.

Bibliographic coupling of the 56 journals based on shared citations revealed six clusters (Figure 3). One (green) centred on *Journal of Feline Medicine and Surgery* and focused on questions of disease, health and management options. A second (purple) had its largest node centred on *Journal of the American Veterinary Medical Association (JAVMA),* where a wide diversity of topics across disease, welfare and management were considered. Wildlife considerations predominated in the diffuse cluster of red journals, without a single journal being prominent. Welfare issues characterised a cluster of journals (blue) around the *Journal of Applied Animal Welfare Science* and, to a lesser extent, *Preventive Veterinary Medicine*. A small cluster (turquoise) developed around *Anthrozoos* and the *Canadian Veterinary Journal*; these journals covered human dimensions of cat management. The final, smallest cluster (olive) centred on the OA journals *Animals* and *Frontiers in Veterinary Science*, where recent papers, including a special issue in *Frontiers in Veterinary Science*, have reported the outcomes of TNR. 

**Figure 4:**
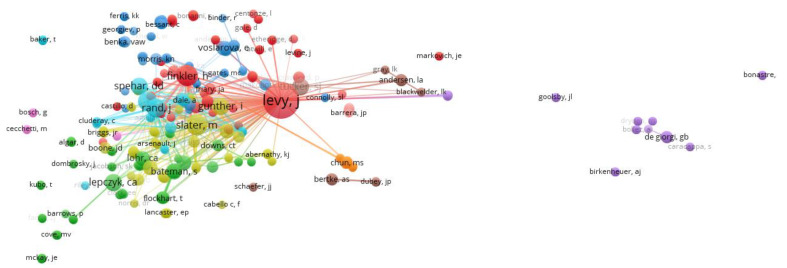
Networks for 398 authors publishing on TNR cat management showing bibliographic coupling links between authors. Here, each node (circle) represents an author. The size of the node represents the number of papers published by each author and the thickness of the lines represents bibliometric coupling, where the strength is determined by the number of shared citations between authors.

Networks based on authors were complex, but five major clusters can be identified (Figure 4). One cluster (red), centred on Levy, covers a range of empirical studies on applications of TNR and management of cats in shelters. A second (light blue), with Rand, Spehar and Wolf as important authors, presents empirical studies supportive of TNR. Support for TNR is also evident in a third cluster (olive) in which Slater is prominent, but they do not extensively cross-cite with the light blue supportive cluster. More cautious or critical views of TNR with concerns for wildlife conservation and public health characterise the fourth group (green), in which Lepczyk is prominent. The final major cluster in which Voslarova and Benka are prominent (dark blue) has concerns with management of cats in shelters and non-surgical contraception. 

### 3.3. Authorship and Journal Analysis

**Figure 5:**
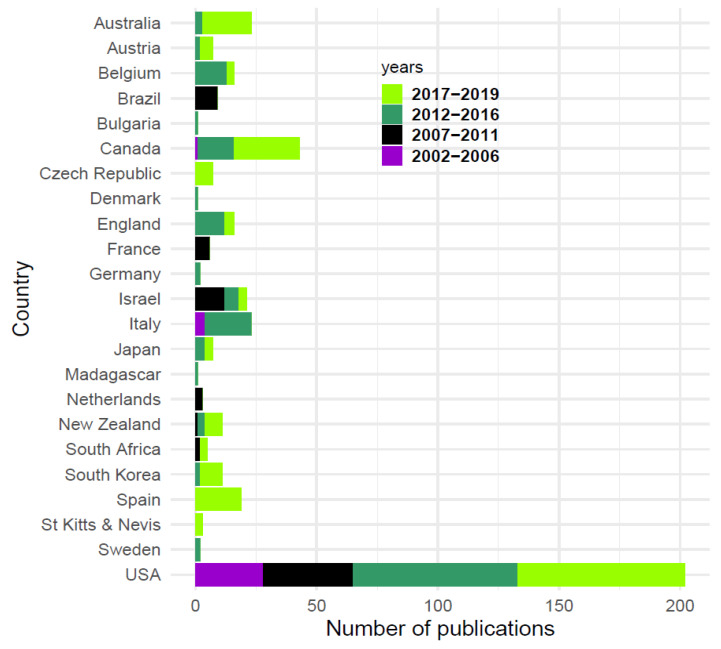
Country of origin of authors publishing TNR papers 2002–2019.

Authors came from 24 different countries, with most from the USA (Figure 5). However, over time, the percentage of USA authors relative to other countries combined declined significantly from 85% in 2002-2006 to 58% in 2017-2019 (*χ^2^*_3_ = 26.380, *p* < 0.0001), with growing numbers of authors from Australia, Canada and Spain. The first international collaborations (where authors on a single paper come from more than one country) appeared in 2012. Overall, five papers included authors from more than one country and were classed as international. 

Across the period 2002–2019, academic authors remained the largest category (Table 1), and they were represented in similar proportions in each time period (*χ^2^*_6_ = 6.93, *p* = 0.33).

TNR papers were published in a total of 56 journals. While the range of journals publishing TNR studies has grown steadily, five journals (*Journal of Feline Medicine and Surgery*, *Journal of the American Veterinary Medical Association (JAVMA)*, *Animals*, *Frontiers in Veterinary Science* and *Journal of Applied Animal Welfare Science*) published nearly half (46%) the contributions. Two of these journals (*Animals* and *Frontiers in Veterinary Science*) are OA, as are a further 10 journals publishing fewer TNR papers (Table 2). Overall, 26% of the papers (38 papers from 12 OA journals, plus one paper published OA in a subscription journal) were published OA, which maximises the availability of this work of public interest. 

### 3.4. Influential Publications

Almost all papers were cited at least once, with only 10% of papers (all in the year range 2017-2019) yet to be cited (probably because of insufficient time since publication for citations to accumulate) (Table 3). 

**Figure 6:**
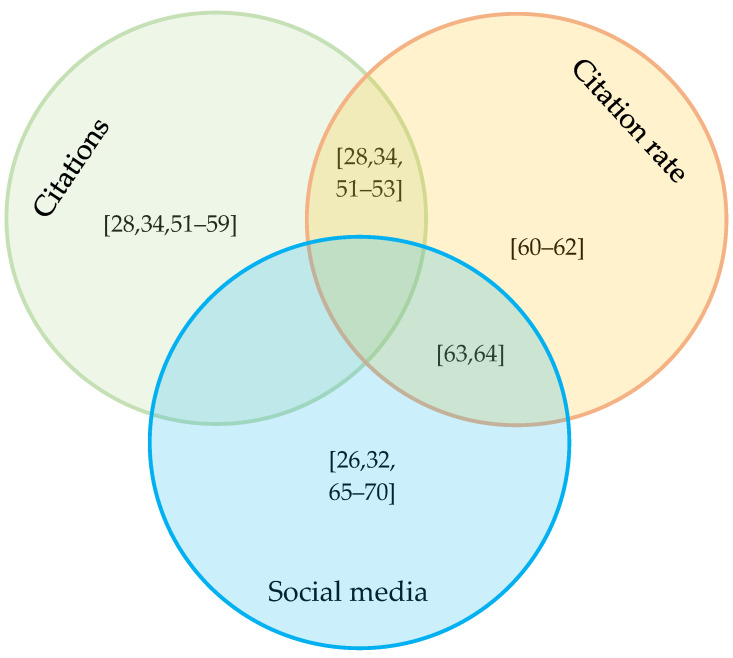
A Venn diagram to represent the top 10 papers on the TNR topic determined by each of citations (total citations received) [28,34,51,52,53,54,55,56,57,58,59], citation rate (citations/year) [28,34,51,52,53,60,61,62,63,64], or social media mentions [26,32,63,64,65,66,67,68,69,70]. Note that the citations list actually includes 11 papers, because two papers tied for tenth spot on the list. The numbers in parentheses correspond to references, the full details of which can be found by number in the reference list at the end of the paper. The Venn diagram indicates all the papers in each list within the respective circles. Papers listed in the overlap regions between circles are in common to two lists: five are in common between the citations list and the citation rate list, and two are in common between the citation rate list and the social media list. No papers are in common to all three lists.

Although we intended to identify the 10 most highly cited papers, two papers tied for the 10th spot, so 11 papers from five journals were represented, ranging from 57–120 citations. These papers were contributed by 30 USA authors, seven Italian authors and three Dutch authors. There were 30 academic authors, seven government authors and three NGO authors. None of these top 11 papers was published OA (Table 4). When citations were corrected for time since publication, eight journals were included in the top 10. Citations/year for the top 10 papers ranged from 5.6 to 8.0. USA authors were the most numerous (24), but there were also five Australian authors and three from the Netherlands. Twenty-three authors were academics, eight were from NGOs and one was from government. Four of the papers were published OA (Table 4). The top 10 papers based on social media ranged from 100–3422 records. They were published in four journals by a total of 23 USA authors and six Australian authors. Ten authors were academics, 18 were affiliated with NGOs and one was from a government organisation. All 10 papers were published OA (Table 4). Comparing the papers on each list, five papers appeared in both the lists for citations and for citations corrected for time since publication. Only two papers were shared between the list for corrected citations and the list for social media mentions. No papers were shared between the citations list and the social media list, so no paper appeared on all three lists (Figure 6). 

The proportion of OA papers in the lists of top ranked papers by citations, citations corrected for time since publication and social media mentions varied significantly, with OA papers predominating in the ranking by social media mentions (two-tailed Fisher exact test, p < 0.01). The proportion of academic authors relative to NGO and government authors combined also varied significantly across the three ranking tables, with NGO and government authors more strongly represented in the social media ranking (*χ^2^*_4_ = 28.25, *p* < 0.01). USA authors predominated on all three lists, with no variation across them in the proportions of countries of authorship (*χ^2^*_2_ = 0.21, *p* = 0.90).

### 3.5. Specific Topics Across All TNR Studies and Assessments of TNR Utility

**Figure 7:**
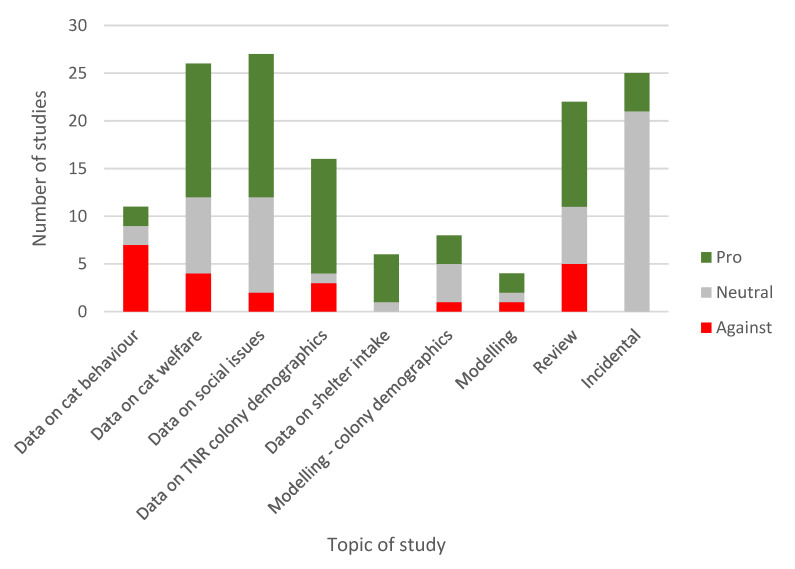
Number of TNR studies in each of nine topics that were positive, neutral or negative in relation to TNR.

Nine broad topics were identified (Figure 7). Twenty-five studies (17%) were in the ‘Incidental’ category, including studies that had used animals sourced through TNR studies (e.g., to obtain ectoparasites) but were not a direct reflection of the practice itself; most of these studies were neutral with regard to TNR aims, although some made comments endorsing TNR. All other topics explicitly dealt with an aspect of the application or evaluation of TNR: data on cat behaviour (8% of studies); data on cat welfare (18%); data on social issues (19%); data on TNR colony demographics (11%); data on shelter intake (4%); modelling colony demographics (6%); modelling (3%); review (15%). The relative proportions of pro and neutral/against stances varied significantly by topic (*χ^2^*_8_ = 23.20, *p* < 0.05). Positive stances were more common for five of the nine categories, most strongly in studies of the influence of TNR on shelter intake and in studies of the demographics of TNR colonies. Negative stances were most prominent for cat behaviour studies. 

Finally, attitude to the use of TNR was associated significantly with whether or not a paper was published OA (*χ^2^*_1_ = 12.66, *p* < 0.01). Papers published OA were more likely to take a positive attitude (pro) towards TNR (Table 5).

## 4. Discussion

### 4.1. Are There Citation Networks in the TNR Literature?

What does a citation analysis reveal about science? Is it only scientists who care about such analyses? MacRoberts and MacRoberts [5] highlight two differing views. First, citations are to acknowledge and give credit to influences. Consequently, citation analyses can reveal where influences or unacknowledged biases lie by showing exchanges between topics, journals or authors. Second, authors cite to *‘contextualize, persuade, convince, back up, supplement, reinforce, support knowledge claims, and display erudition’* with any acknowledgement of influence *‘merely an inadvertent side effect of trying to convince the audience that the author is expert in the subject’* ([5], p.478). It follows that, if the first view applies, citation networks should track the chain of influences behind studies and should not reveal closed enclaves of citations. However, if the second view applies, then citation networks are more likely to display closed communities of common interest. Thus from the perspective of all stakeholders, management guided by narrow aspects of this TNR literature could be compromised by closed and self-reinforcing viewpoints. 

Our examination of key terms around TNR cat management indicates that there are five main groups of publications on the topic: 1. disease and health, 2. free-roaming cats, 3. feral and domestic cat management, 4. population control, and 5. wildlife and predation. These papers represent different topics or angles on the debate, approximating the interest groups of veterinarians (topics 1 and 4) the public, animal protection/welfare advocates (topics 2 and 3), and conservation advocates (topics 4 and 5). All these interest groups are identified by Farnworth et al. [71] as important stakeholders in the management of invasive species. The topics that we identified parallel those central to papers with high citations or high social media mentions, further emphasising the prominence of these topics in TNR publications. 

Citation groups also formed around journals and authors. In some cases, these reveal clear professional interests, such as veterinarians reading and publishing in *JAVMA* or, if they are feline specialists, in *Journal of Feline Medicine and Surgery*. Similarly, prolific authors such as Levy, Slater, Lepczyk and Rand are nodes of citation for others contributing to the TNR literature.

There are also signs in the citation networks suggesting problems might develop in terms of communication across the full spectrum of the TNR literature. For example, the three largest nodes in the key terms analysis were ‘domestic cats’, ‘free-roaming cats’, and ‘feral cats’; but they fell into different clusters. ‘Free-roaming cats’ were perceived as distinct from the other cat categories. The language used to describe cats may have consequences for how they are perceived that ultimately translates into how they are managed [72,73], so these tendencies to separate categories of cats by their situation/location may influence how people search the literature and how findings are applied. The nodes that develop around authors and journals may also indicate practices for searching the literature (e.g., citation alerts for new papers by a specific author) that could contribute to the development of closed groups, reinforcing specific internal views without challenge from other interpretations. Variation in terminology is a significant issue in managing cat populations [31,74], which, in turn, is challenging for developing bibliometric analyses. 

Misemer et al. [6] noted the negative consequences that can arise if citation networks become closed. In particular, reliance on citation webs and failure to review literature systematically could combine with rapid growth in publications to reinforce conscious or unconscious biases in referencing. Greenberg ([75], p.7) noted *‘... the* *power of citation through the choice of which papers to cite and which to ignore (citation bias), by citing but distorting content (citation diversion), and by using citation to invent fact (citation transmutation, dead end citation, and back door invention).’* We believe we have shown the potential for such biases to develop in the TNR literature. This could lead to sub-optimal management responses, especially if decision-makers are operating within a closed citation network. To address this problem, we suggest that editors of journals publishing on TNR cat management could aim to broaden scientific debate on the topic by:

- Selecting reviewers who have complementary expertise; i.e., what Fletcher and Black ([9], p. 522) called *‘Choose reviewers for all the manuscript’s agendas’* (e.g., a paper arguing for lethal control of cats from a human health perspective will benefit from review from an animal welfare perspective) to generate a greater depth of scientific debate around the topic. Sourcing reviewers from the reference list of submitted papers would be problematic, as would be accepting all the authors’ suggestions for reviewers.
- Having stringent requirements to declare and publish financial conflicts of interest, insisting that authors include a section on the limitations of their study, querying if employers or funders restricted what could be published, and ensuring that the power balance in negotiations over revisions remains with the editors, not the authors [9].
- Publishing reviews and responses alongside the paper in cases of unresolved differences of opinion between reviewers and authors [76].
- Checking review papers to avoid what Greenberg ([75], p. 4) called a lens effect, *‘in which a small number of these influential review papers and model papers containing no data on claim validity collected and focused citation (similar to a magnifying lens collecting light) on particular primary data papers supportive of the belief, while isolating others that weakened it.’*
- Checking abstracts thoroughly to ensure that they are an accurate reflection of the content of the paper [77].
- Favouring systematic reviews that document clearly how the review was conducted, with decision rules for including or excluding specific studies [78], over descriptive reviews that may be selective in the literature included without revealing the reasons.

### 4.2. What are the Prominent Issues in TNR Management?

The TNR papers that have a high profile through either citations or social media indicate the central issues in the influential TNR literature. Citations reveal academic interest and use, while social media mentions are perhaps a better indication of the matters that occupy public attention. 

Excepting a small handful of very recent publications that have not had an opportunity to accrue citations, all TNR papers have been cited. The most highly cited papers, either by the total number of citations or as citations per year, indicate the range of concerns around TNR, including: human dimensions of people’s interactions with cats [56], the evaluation of TNR programs by empirical studies [51,64], critiques of evidence and interpretations [28], disease transmission (both in terms of cat welfare [53] and human health [58]) and reviews of control options [34]. Critics of citation analysis note that many citations may be perfunctory or redundant, as opposed to significant ones that stimulate a new idea, provide a new method, or otherwise provide an integral part of the citing paper [79]. Authors may not even cite key influences on their work at all because they are not in a convenient citable form or because of ‘traditional non-citation’ for some sources such as established experimental or analytical techniques [5], or exercise conscious or sub-conscious bias in selecting studies to cite (for example, a tendency to cite positive studies more than negative ones is noted in fields as diverse as ecotoxicology [7], diagnostic imaging [80], and biomedical science [6,8]). Thus, the high citations reported are indicative of use, but not necessarily of quality. 

Citation data note only the use of publications for academic purposes and may not measure other influences, such as the extent to which papers may be consulted but not subsequently cited, or the uptake of the work by news outlets, practitioners who do not publish, or the public [81,82]. Alternative metrics may be more useful in this regard. However, as is the case with scientific citations, social media mentions are not necessarily an indication of quality. Alternative metrics do, however, confirm public interest in a topic and, potentially, wide awareness. The data on the use of TNR papers in social media provided a revealing contrast to the citation-based assessments, with only one of the highly cited papers [64] appearing in the top 10 papers as ranked by social media mentions. The social media phenomenon is recent, so it is unsurprising that the most highly ranked papers on this measure have all been published since 2014.

Overall, the 25 unique papers that featured on one or more of the lists of influential papers included 17 papers taking a positive view of TNR, four that were neutral and four that were negative. This indicates a diversity of opinion at the influential level, but that most influential papers are positive towards TNR. A detailed meta-analysis would be needed to discriminate whether this is a true reflection of the data in the field, or an example of bias towards positive findings.

Considering all the studies rather than just those ranked highly, studies taking positive and negative attitudes to TNR were present in all topics identified (except the ‘incidental’ topic where most studies were neutral and the ‘shelter intake’ topic), indicating that divergent opinions are being expressed. Whether these opinions are being considered by the opposite camps is not revealed by our study. Positive studies were more likely to be published OA, so they may have a higher profile in the broader community. 

### 4.3. Who Publishes on TNR and Where Do They Publish?

Since the first TNR papers were published in 2002, the literature remains dominated by academic authors. This is perhaps a logical extension of its connection to veterinary expertise, although academics from other disciplines also contribute. The imperative to publish for academic career advancement may also encourage substantial contributions from academic authors [83]. However, academic authors do not have a monopoly on papers enjoying high citations or high media counts, so the contributions from government or NGO authors are clearly significant. 

While most TNR authors in the early years came from the USA, this predominance has reduced significantly over time, with growing contributions from 23 other countries. The breadth of international authorship shows the extent of problems with unowned cats internationally. A challenge is to evaluate robust findings that are likely to translate to many localities, as distinct from those that may have more limited regional application (e.g., see [30,31] cf. [33]). 

Open Access was the choice for authors of 26% of the papers in our sample, with two OA journals (*Animals* and *Frontiers in Veterinary Science*) just behind two veterinary subscription journals (*Journal of Feline Medicine and Surgery* and *Journal of the American Veterinary Medical Association (JAVMA)*) in numbers of TNR papers published. There is extensive and on-going debate as to whether or not papers available by OA are more highly cited than those that are not (e.g., [84,85,86]). Irrespective of that debate, it seems that when awareness on social media is concerned, OA is a significant advantage. All 10 papers ranked most highly on the basis of social media communications are available by OA, as opposed to none on the most highly cited list and four on the list of high citation rate. Certainly, the promotion of publications on social media is substantially easier when they are available by OA, which maximises the availability of this work of public interest.

### 4.4. Strengths and Limitations of the Study

This is a descriptive study, so it includes no assessment of the validity of the findings of any of the TNR papers in the sample. The literature sample was based on WoSCC, which is a selective database and will have missed some TNR studies, especially those published in languages other than English [87]. Like any database, it is also potentially subject to errors or to missing data [79]. The use of citations as a measure of publication influence is biased towards older papers, which have more time to accrue citations (although we attempted to balance this by including citation rate). Furthermore, citation frequency is often biased towards positive studies [6], as studies in numerous fields confirm [7,8,88], and therefore is not necessarily a sign of quality. The use of social media indicators is similarly biased, although towards newer papers. The uses of alternative metrics are, therefore, only valid indicators of the impact of recent studies (e.g., Twitter launched in 2006 and only began to grow rapidly from 2007 [89]). The modest sample size of the TNR literature, especially with many recent papers that take time to accrue citations, may also exaggerate some effects that might be moderated in a larger literature.

Despite these limitations of citation and alternative metric measures, this study offers specific strengths. While WoSCC is selective, it excludes studies published in potentially questionable journals. Examining the topics prominent in papers with a high profile by both citation and social media measures, coupled with descriptions of networks based on keyword analysis, gives a triangulated view of important topics in the TNR literature.

## 5. Conclusions

The growth in the TNR literature indicates global concern to address the problems of stray cat population control, their interactions with wildlife, and disease transmission (including implications for pet cats, wildlife and humans). Network analysis in the TNR literature drawing on the journals publishing the work and the connections between authors and journals established through referencing indicates the potential for self-reinforcing groups of authors to form. While, on the one hand, it is encouraging that diverse views are expressed, there is a risk of lack of dialogue between interest groups on the other hand. This could reduce the opportunity for constructive exchanges of differing views to refine arguments, exchange information, or develop research programs to provide empirical data for dispute resolution. This could compromise management of free-roaming cats that addresses the concerns of all stakeholders. Journal editors could encourage such exchanges by choosing reviewers from different camps to assess manuscripts and by asking authors to acknowledge alternative views.

## Figures and Tables

**Table 1:** Affiliations of authors publishing TNR papers, 2002–2019.

Affiliation	2002–2006	2007–2011	2012–2016	2017–2019 *	Totals
Non-government organization/private researcher	5	8	29	46	88 (18.5%)
Government	1	2	12	15	30 (6.3%)
Academia	27	60	113	158	358 (75.2%)
Total authors	33 (6.9%)	70 (14.7%)	154 (32.3%)	219 (46.0%)	476

* Papers were included up to 13 January 2020.

**Table 2:** Journals publishing TNR papers, 2002–2019.

Journal	Number of Papers/Journal
*Journal of Feline Medicine and Surgery, Journal of the American Veterinary Medical Association (JAVMA)*	17
Animals *	13
Frontiers in Veterinary Science *	11
*Journal of Applied Animal Welfare Science*	10
*Preventive Veterinary Medicine*	4
Animal Conservation, Anthrozoos, Canadian Veterinary Journal, Conservation Biology, Journal of Wildlife Management, PeerJ *, PLOS One *, The Veterinary Journal **, Urban Ecosystems, Veterinary Parasitology, Zoonoses and Public Health	3
*Applied Animal Behaviour Science, Reproduction in Domestic Animals, Wiener Tierarztliche Monatsschrift, Wildlife Research*	2
Acta Veterinaria Brno *, American Journal of Reproductive Immunology, American Journal of Veterinary Research, Animal Welfare, Annali dell’Istituto Superiore di Sanita *, Biological Conservation, Biological Invasions, BioMed Research International *, British Journal of Nutrition, Canadian Journal of Veterinary Research, Dialogue-Canadian Philosophical Review, Ecology and Evolution *, Ecological Modelling, Ecology and Society *, Global Ecology and Conservation *, Human-Wildlife Interactions, Italian Journal of Zoology, Journal of Mammalogy, Journal of Veterinary Behaviour, Journal of Veterinary Internal Medicine *, Mammal Study, Natural Areas Journal, Pacific Science, Physiology and Behavior, Puerto Rico Heath Sciences Journal *, Revista Medica De Chile, Southeastern Naturalist, Social and Cultural Geography, Theriogenology, Topics in Companion Animal Medicine, Vector-Borne and Zoonotic Diseases, Veterinary Clinics of North America - Small Animal Practice, Veterinary Dermatology, Veterinary Record, Vlaams Diergeneeskundig Tijdschrift	1

* Open Access journal. ** A paper was published OA in this subscription journal.

**Table 3:** Proportion of TNR papers cited by year of publication.

Year Range	Number of Papers	Proportion Cited
2002–2006	16	1
2007–2011	26	1
2012–2016	43	1
2017–2019 *	63	0.78

* Papers were included up to 13 January 2020.

**Table 4:** The top TNR papers between 2002–2019 ranked on citations (top 10), citations/year since publication (top 10), or social media mentions (top 10) since publication.

Ref	Title (Category of Study)	Year	Journal	Author Country	Author Classification	Stance Taken	Citation Ranking (Citations Count)	Citation Rate Ranking (Citations/Year)	Social Media Ranking (Social Media Mentions)
[53]	Prevalence of infectious diseases in feral cats in Northern Florida (Data on cat welfare)	2004	*Journal of Feline Medicine and Surgery*	8 USA	8 academic	Neutral	1 (120)	2 (7.5)	
[51]	Evaluation of the effect of a long-term Trap–Neuter–Return and adoption program on a free-roaming cat population (Data on TNR colony demographics)	2003	*JAVMA*	3 USA	1 academic, 2 NGO	Pro	2 (96)	8 (5.6)	
[28]	Critical assessment of claims regarding management of feral cats by Trap–Neuter–Return (Review)	2009	*Conservation Biology*	3 USA	2 academic, 1 NGO	Against	3 (88)	1 (8)	
[57]	Humane strategies for controlling feral cat populations (Review)	2004	*JAVMA*	2 USA	2 academic	Pro	4 (78)		
[56]	Characteristics of free-roaming cats and their caretakers (TNR colony demographic)	2002	*JAVMA*	2 USA	2 academic	Pro	5 (75)		
[34]	A review of feral cat control (Review)	2008	*Journal of Feline Medicine and Surgery*	1 USA	1 academic	Pro	6 (74)	6 (6.2)	
[55]	Reproductive capacity of free-roaming domestic cats and kitten survival rate (Data on cat welfare)	2004	*JAVMA*	3 USA	3 academic	Neutral	7 (71)		
[54]	Management of feral domestic cats in the urban environment of Rome (Italy) (Data on TNR colony demographics)	2006	*Preventive Veterinary Medicine*	7 Italian	6 government, 1 academic	Neutral	8 (63)		
[52]	Estimation of the dietary nutrient profile of free-roaming feral cats: possible implications for nutrition of domestic cats (Incidental)	2011	*British Journal of Nutrition*	3 Netherlands authors	3 academic	Neutral	9 (57)	3 (6.6)	
[58]	Outdoor fecal deposition by free-roaming cats and attitudes of cat owners and nonowners toward stray pets, wildlife, and water pollution (Data on cat welfare)	2006	*JAVMA*	5 USA authors	4 academic, 1 government	Against	10 (57)		
[59]	Survival, fecundity, and movements of free-roaming cats (Data on stray cat behaviour)	2007	*Journal of Wildlife Management*	3 USA authors	3 academic	Against	10 (57)		
[32]	A long-term lens: cumulative impacts of free-roaming cat management strategy and intensity on preventable cat mortalities (Modelling of colony demographics)	2019	Frontiers in Veterinary Science *	8 USA	6 NGO, 1 academic, 1 government	Pro			8 (352)
[65]	Cat gets its tern: a case study of predation on a threatened coastal seabird (Data on cat behaviour)	2019	Animals *	3 Australian	3 academic	Against			1 (3422)
[66]	Study of the effect on shelter cat intakes and euthanasia from a shelter neuter return project of 10,080 cats from March 2010 to June 2014 (Data on shelter intake)	2014	PeerJ *	2 USA	2 NGO	Pro			2 (3032)
[62]	Long-term fertility control in female cats with GonaCon ™, a GnRH immunocontraceptive (Incidental)	2011	*Theriogenology*	4 USA	3 academic, 1 government	Pro		10 (5.4)	
[64]	Effect of high-impact targeted Trap–Neuter–Return and adoption of community cats on cat intake to a shelter (Data on shelter intake)	2014	The Veterinary Journal **	3 USA	3 academic	Pro		4 (6.5)	4 (1310)
[67]	Cat colony caretakers’ perceptions of support and opposition to TNR (Data on social issues)	2019	Frontiers in Veterinary Science *	3 Australian	3 academic	Pro			10 (108)
[63]	An examination of an iconic Trap–Neuter–Return program: The Newburyport, Massachusetts case study (TNR colony demographics)	2017	Animals *	2 USA	2 NGO	Pro		7 (5.7)	6 (731)
[26]	A case study in citizen science: the effectiveness of a Trap–Neuter–Return program in a Chicago neighborhood (TNR colony demographics)	2018	Animals *	2 USA	2 NGO	Pro			5 (882)
[68]	The impact of an integrated program of return-to-field and targeted Trap–Neuter–Return on feline intake and euthanasia at a municipal animal shelter (TNR colony demographics)	2018	Animals *	2 USA	2 NGO	Pro			9 (252)
[69]	Integrated return-to-field and targeted trap-neuter-vaccinate-return programs result in reductions of feline intake and euthanasia at six municipal animal shelters (TNR colony demographics)	2019	Frontiers in Veterinary Science *	2 USA	2 NGO	Pro			3 (1797)
[60]	Application of a protocol based on Trap–Neuter–Return (TNR) to manage unowned urban cats on an Australian university campus (TNR colony demographics)	2018	Animals *	2 Australian	2 NGO	Pro		9 (5.5)	
[61]	Trap–Neuter–Return activities in urban stray cat colonies in Australia (Data on social issues)	2017	Animals *	3 Australian	2 academic, 1 NGO	Pro		5 (6.3)	
[70]	The road to TNR: examining Trap–Neuter–Return through the lens of our evolving ethics (Review)	2019	Frontiers in Veterinary Science *	2 USA	1 NGO, 1 academic	Pro			7 (371)

* Open Access. ** Paper published OA in a subscription journal.

**Table 5:** Attitudes to TNR in papers published OA and non-OA formats.

Attitude to TNR	Published OA	Not published OA	Total
Positive	26	42	68 (46.9%)
Neutral	8	46	54 (37.2%)
Negative	2	21	23 (15.9%)
Total	36 (24.8%)	109 (75.2%)	145 (100%)
